# Novel Cyclic Peptide–Drug Conjugate P6-SN38 Toward Targeted Treatment of EGFR Overexpressed Non-Small Cell Lung Cancer

**DOI:** 10.3390/pharmaceutics16121613

**Published:** 2024-12-19

**Authors:** Andrii Bazylevich, Ayala Miller, Iryna Tkachenko, Maia Merlani, Leonid Patsenker, Gary Gellerman, Bat Chen R. Lubin

**Affiliations:** 1 Department of Chemical Sciences, Ariel University, Ariel 40700, Israel; andriib@ariel.ac.il (A.B.); irynat@ariel.ac.il (I.T.); leonidpa@ariel.ac.il (L.P.); garyg@ariel.ac.il (G.G.); 2 Department of Chemical Engineering, Biotechnology and Materials, Ariel University, Ariel 40700, Israel; 3 Agriculture and Oenology Department, Eastern Regional R&D Center, Ariel 40700, Israel; 4 I. Kutateladze Institute of Pharmacochemistry, Tbilisi State Medical University (TSMU), Vashlijvari 0159, Georgia

**Keywords:** EGFR, NSCLC, targeted cancer therapy, peptide–drug conjugate, topo I inhibitor, SN38

## Abstract

**Background/Objectives:** Here, we report on the synthesis and biological evaluation of a novel peptide–drug conjugate, P6-SN38, which consists of the EGFR-specific short cyclic peptide, P6, and the Topo I inhibitor SN38, which is a bioactive metabolite of the anticancer drug irinotecan. **Methods:** SN38 is attached to the peptide at position 20 of the E ring’s tertiary hydroxyl group via a mono-succinate linker. **Results:** The developed peptide–drug conjugate (PDC) exhibited sub-micromolar anticancer activity on EGFR-positive (EGFR+) cell lines but no effect on EGFR-negative (EGFR−) cells. In vivo studies have shown that this PDC specifically accumulates in EGFR+ non-small cell lung cancer (NSCLC) xenografts and presents superior anticancer activity compared to the EGFR-specific antibody cetuximab (Erbitux^TM^) and free SN38. The 10 mg/kg dose of P6-SN38 in a side-by-side EGFR+/EGFR− xenograft shows eradication of the EGFR+ tumor with good tolerance, but no inhibition of tumor growth of the EGFR− counterpart. **Conclusions:** The PDC examined in this study was proven to be highly efficient for NSCLC, broadening its utilization for targeted cancer therapy in EGFR overexpressed cancers.

## 1. Introduction

Non-small cell lung cancer (NSCLC) is one of the most prevalent tumors, associated with high morbidity and mortality [1]. Drug resistance in NSCLC is a significant cause of therapeutic failure [2], leading to a less than 15% five-year survival rate [3]. Current standard treatment involves surgical resection followed by chemotherapy and radiotherapy [4,5]. Combination therapy strategies and the use of drug cocktails enhance the overall five-year survival rate and improve the patient’s quality of life [6]. Hence, novel drug candidates having limited treatment-related side effects and overcoming drug resistance are urgently required.

One of the promising approaches developed in recent years to improve chemotherapy outcomes and reduce side effects involves targeting the mutated epidermal growth factor receptor (EGFR) [7]. Recently developed personalized therapy with tyrosine kinase inhibitors (TKIs) of EGFR has changed the treatment options for NSCLC [8,9]. Nevertheless, although these medications initially produce a favorable response in many patients, the majority ultimately develop drug resistance, which represents a significant limitation that undermines the long-term effectiveness of the treatment [10,11]. Higher survival rates were seen in individuals with advanced lung cancer who received targeted therapy using anti-EGFR antibodies, such as cetuximab (Erbitux) and amivantamab (Rybrevant), in combination with conventional chemotherapy [12,13].

Among the diverse range of targeted therapeutic approaches, peptide–drug conjugates (PDCs) are being investigated as a promising strategy for the selective delivery of cytotoxic agents to cancer cells [14]. Certain receptors essential for cancer cell survival and growth are overexpressed in these cells. By utilizing peptide carriers as ligands for these overexpressed receptors, PDC technology leverages this phenomenon [15,16]. This approach holds the potential to specifically target cancer cells while reducing off-target side effects, thereby substantially expanding the therapeutic window and underscoring the importance of advancing their development [17].

In general, peptide–drug conjugates (PDCs) offer advantages over other targeted therapies, such as antibody–drug conjugates (ADCs), particularly in terms of enhanced tumor penetration and their straightforward, efficient synthesis [18]. Nevertheless, PDCs, namely linear ones, may have drawbacks such as low bioavailability because of peptide structural disintegration and quick renal clearance associated with their smaller size. Structural alterations, in particular cyclization [19,20,21], can address some of these difficulties, making PDCs excellent carriers for targeted drug delivery.

So far, two peptide–radionuclide conjugates, 177Lu-DOTA-TATE and 177Lu-PSMA-617, have been approved for clinical use to treat somatostatin receptors and prostate-specific membrane antigen (PSMA) overexpressing cancers, respectively [22,23]. Given the recognized importance of PDCs for cancer therapy, we propose here to utilize short cyclic peptides as drug carriers for targeted treatment of NSCLC.

Several EGFR-binding cyclic peptides have been developed in the past as potential carriers for targeted therapy and tumor imaging [24]. Other EGFR-specific peptides were explored in human hepatoma cells, human chronic myeloid leukemia cells, and murine neural stem cells involved in nerve regeneration [25]. In our previous work, we reported on a series of EGFR targeting disulfide-bridged cyclic peptides that can selectively bind and internalize into wild-type and mutant EGFR overexpressed NSCLC cells [26]. The lead peptide carrier, P6, was found to be the most selective drug carrier in vitro. In this work, we have studied both in vitro and in vivo therapeutic potential of the newly developed peptide–drug conjugate P6-SN38, comprised of the cyclic peptide carrier P6 and the potent Topo I isomerase inhibitor SN38, the active metabolite of Irinotecan [27,28], on the EGFR+ NSCLC model.

**Figure 1:**
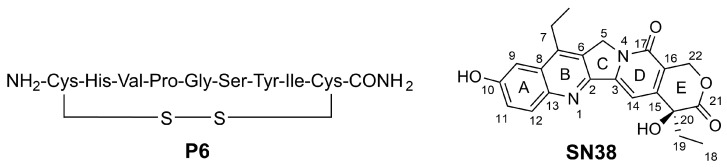
Structure of P6 and SN38. To present the locations of the functional groups more clearly, the key positions of the SN38 structure were numbered.

The conjugation of the SN38 to the carrier is usually performed either through the tertiary alcohol group at position 20 of the lactone ring E [29,30] or via the phenolic hydroxyl group at position 10 of the quinoline ring A of the drug [31,32] (Figure 1).

The SN38 hydroxyl group enables linkage through cleavable functionalities like ester [33,34], carbonate [29], and carbamate [30]. The pharmacokinetics, release profile, and stability of the drug are all significantly affected by the attachment of SN38 to a carrier molecule through these chemical groups. Because of its adequate liability in a biological environment, we opted to conjugate SN38 through the tertiary alcohol group by ester linkage, which permits the payload to be released at the target site [35]. This approach results in a more stable tertiary ester linkage, effectively shielding the ester bond from nucleophilic attack [36]. Additionally, conjugation at this site stabilizes the lactone ring of the drug, which is highly susceptible to hydrolysis at physiological pH of about 7.4 [37]. Notably, the conjugation of SN38 to the carrier via the tertiary alcohol group of the lactone ring requires prior protection of the phenolic hydroxyl group, typically by using silyl ether or Boc groups [34].

The implementation of the aforementioned techniques and factors resulted in the development of a novel P6-SN38 PDC. It consists of a disulfide-bridged cyclic nonapeptide P6 conjugated to SN38 through the succinate (suc) ester linkage at the tertiary alcohol group of the lactone ring of the drug. In this research, P6-SN38 was prepared and subsequently assessed in vitro and in vivo for treating EGFR overexpressed non-small cell lung cancer.

## 2. Materials and Methods

### 2.1. General

LC/MS assessment was conducted using an Agilent Technologies 1260 Infinity (LC) coupled with a 6120 quadrupole (MS), employing an Agilent SB-C18 column (1.8 µm, 2.1 mm × 50 mm) maintained at 50 °C. The mobile phase consisted of water-acetonitrile (ACN) with 0.1% formic acid. High-resolution mass spectrometry (HRMS) was performed in positive ion mode using an Agilent 6550 iFunnel Q-TOF LC/MS system (Eldan Electronic Instruments Ltd., Petah Tikva, Israel). Proton (^1^H) and carbon (^13^C) NMR spectra were recorded in CDCl_3_ at 300 K on a Bruker Avance III HD spectrometer (^1^H at 400 MHz, ^13^C at 100 MHz) equipped with a BBO probe and a Z-gradient coil. Flow cytometry analysis was performed on a Beckman CytoFLEX V5-B4-R3 flow cytometer (Bio-Rad Laboratories, Inc., Rishon Le Zion, Israel), with data processed using FlowJo software (V9). In vivo imaging was carried out using the IVIS Spectrum multispectral fluorescence system (PerkinElmer, Waltham, MA, USA). P6-SN38 PDC was purified by preparative HPLC (ECOM), utilizing dual UV detection (254 and 214 nm). A Phenomenex^®^ 10 µm RP18 column (250 mm × 21.2 mm) was employed, with the column maintained at ambient temperature. Eluents A (0.1% formic acid in water) and B (acetonitrile) were used in a gradient from 100% A to 100% B over 35 min at a flow rate of 25 mL/min. Chemical reactions were monitored by thin-layer chromatography (TLC) on Merck Silica Gel 60 F-254 and LC/MS.

### 2.2. Reagents and Standard Samples

All solutions for preparative purification were prepared using Milli-Q water (Millipore Corporation, Bradford, MA, USA). Tryptone, yeast extract, Na_2_HPO_4_ (disodium hydrogen phosphate), KH_2_PO_4_ (potassium dihydrogen phosphate), Xgal (5-bromo-4-chloro-3-indolyl-β-D-galactopyranoside), IPTG (isopropyl-β-D-thiogalatopyranoside), CHAPS (3-[(3-cholamidopropyl)dimethylammonio]-1-propanesulfonate), glycine, SDS (sodium dodecyl sulfate), Triton X-100, NaCl, PEG 8000 (polyethylene glycol), agarose, and glycerol were purchased from Alfa Aesar, Israel, or Sigma-Aldrich, Israel. Epidermal growth factor (EGF) was sourced from Abcam (Cambridge, MA, USA). Dulbecco’s Modified Eagle Medium (DMEM), Roswell Park Memorial Institute (RPMI)-1640 medium, trypsin, fetal bovine serum (FBS), XTT cell viability assay kit (2,3-bis-(2-methoxy-4-nitro-5-sulfophenyl)), penicillin, and streptomycin were obtained from Sartorius, Israel. Mouse antibody APC anti-human EGFR, APC goat anti-mouse IgG, cell staining buffer, and DMSO were sourced from ENCO, Petah Tikva, Israel.

### 2.3. Synthesis of the Cyclic P6-SN38

Synthesis of the TBDMS-SN38-suc. To the solution of TBDMS-SN38 (506 mg, 1 mmol, obtained according to the procedure [38]) in anhydrous DCM (25 mL), were added succinic anhydride (150 mg, 1.5 mmol), excess DIPEA (0.5 mL), and DMAP (61 mg, 0.5 mmol). The mixture was stirred for 36 h at r.t. The progress of the reaction was monitored by TLC (MeOH/DCM (1/9, *v*/*v*). Upon completion of the reaction, the DCM solution was washed with ice-cooled 0.01 M HCl, water, and brine. The organic layer was separated, dried over anhydrous Na_2_SO_4_, the solvent was evaporated, and the product was purified by flash chromatography on silica gel 60 using MeOH/DCM (0.5:9.5, *v*/*v*) to afford 370 mg (62% yield) of TBDMS-SN38-suc as a light-yellow solid. ^1^H NMR (400 MHz, CDCl_3_) δ, ppm: 7.80 (d, J = 9.2 Hz, 1H), 7.32 (s, 1H), 7.23 (d, J = 2.4 Hz, 1H), 7.19 (dd, J = 2.8, 6.6 Hz, 1H), 5.71 (d, J = 16.8 Hz, 1H), 5.37 (d, J = 16.8 Hz, 1H), 5.06 (d, J = 18.4 Hz, 1H), 4.93 (d, J = 18.4 Hz, 1H), 3.01–2.70 (m, 6H), 2.25–2.10 (m, 2H), 1.28 (t, J = 7.7 Hz, 3H), 1.04 (s, 9H), 1.01 (t, J = 7.5, 3H), 0.29 (s, 3H), 0.28 (s, 3H); ^13^C NMR (CDCl_3_) δ, ppm: 174.1, 171.5, 167.7, 157.4, 155.4, 149.2, 146.5, 145.9, 144.6, 144.9, 130.7, 128.2, 127.1, 126.3, 119.4, 110.5, 97.1, 76.4, 67.0, 49.2, 31.6, 29.7, 29.5, 25.8, 23.2, 18.5, 13.7, 7.7, 4.1; HRMS: *m*/*z* calcd. for C_32_H_39_N_2_O_8_Si [M + H]^+^: 607.2476; found 607.2489.

Synthesis of the resin-bound NH_2_-GABA-P6. The synthesis of the peptide P6 and P6 equipped with the GABA linker was described in our previous work [26].

Conjugation of the TBDMS-SN38-suc to the resin-bound NH_2_-GABA-P6. To the reaction vessel containing NH_2_-GABA-P6 [26] (300 mg) immobilized on Rink Amide resin (RA, loading 0.6–0.7 mmol/g, 100–200 mesh, Sigma-Aldrich, Rehovot, Israel), was added a solution containing TBDMS-SN38-suc (97 mg, 0.16 mmol), PyBOP (83 mg, 0.16 mmol), and DIPEA (140 µL, 0.8 mmol) in DMF (peptide grade, 5 mL). The reaction vessel was shaken for 2 h. Then, the peptidyl resin was washed with DMF (3 × 5 min), DCM (3 × 5 min), and cleaved by the cocktail containing 95% trifluoroacetic acid (TFA), 2.5% H_2_O, and 2.5% triisopropylsilane (TIS) for 1 h at RT. After the evaporation of the solvent by the nitrogen stream, the crude product was precipitated by adding cold diethyl ether. The precipitate was collected, dissolved in t CH_3_CN/H_2_O (1:1, *v*/*v*), and purified by preparative HPLC. The pure fractions were combined and lyophilized, giving pure (>95%, LC/MS 254 nm) P6-SN38 (34 mg, yield 21%) as a yellow powder. RT_LC_ = 4.8 min; HRMS: *m*/*z* calcd. for C_72_H_93_N_15_O_19_S_2_^2+^ 767.8107; found: 767.8141 [M + 2H]^2+^.

### 2.4. Cells Lines

Lung cancer (H1299), myeloid leukemia (K562), and embryonic (HEK293) cell lines were acquired from the ATCC (Manassas, VA, USA) and cultured in RPMI 1640 medium supplemented with 10% fetal bovine serum (FBS), 2 mM L-glutamine, and 1% penicillin/streptomycin (all from Biological Industries, Kibbutz Beit Haemek, Israel). The cells were maintained at 37 °C in a humidified atmosphere containing 5% CO_2_.

### 2.5. Cellular Toxicity

Cell proliferation in the presence of P6-SN38 was evaluated using a commercial XTT assay kit (Enhanced Cell Counting Kit 8 (WST-8/CCK8); Elabscience, Houston, TX, USA). A total of 10^4^ cells per well were seeded in 96-well plates and incubated overnight in complete RPMI growth medium. After washing, the cells were cultured in 100 µL of fresh medium containing varying concentrations of free SN38 or PDC for 3 h and 6 h (pre-incubation). Following this, the cells were washed again and incubated for an additional 24, 48, and 72 h. The XTT reagent was then added to each well, and the plate was incubated for 2 h at 37 °C. For cytotoxicity analysis, absorbance was measured at 450 nm and 630 nm using a TECAN Infinite M200 ELISA reader (Citrine Solutions, Oranit, Israel). All assays were performed in triplicate, and each experiment was repeated three times.

### 2.6. Apoptosis Assay with Flow Cytometry

H1299 cells were seeded at the concentration of 5 × 10^4^ cells/mL in the RPMI 1640 growth medium and incubated in 6-well plates at 37 °C under 5% CO_2_. The next day, the medium was removed, and P6-SN38, or free SN38 (2.5 µM), in fresh medium was added and incubated for 6 h. Then, the medium was removed by washing out, fresh medium was added, and the plates were incubated for an additional 24 and 48 h. The cells were collected with 0.25% trypsin/EDTA solution and washed with PBS twice at 1500 rpm for 5 min. After that, Annexin-FITC (5 µM) and propidium Iodide (PI, 5 µM) were added (both from Abcam, Cambridge, UK). The cells were incubated for 15 min in the dark, and the samples (2 × 10^4^ cells) were analyzed by flow cytometry.

### 2.7. Cell Cycle Analysis

Cell cycle analysis was conducted using a cell cycle detection kit (Biotopped, Huangshan, China). H1299 cells were seeded in 5 × 10^4^ cells/mL concentration in the complete growth medium. (RPMI 1640) Incubation was performed in 6-well plates at 37 °C under 5% CO_2_. The next day, the medium was removed, and the fresh medium containing P6-SN38 or free SN38 (2.5 µM) was added and incubated for 6 h. Then, the medium was removed by washing, a fresh medium was added, and the cells were incubated for an additional 24 and 48 h. Then, the cells were trypsinized and washed with PBS twice at 1500 rpm for 5 min. Cells were fixed in 66% ethanol for 2 h. Then, cells were incubated with 0.2 mL buffer solution containing PI (25 μL) and RNase A (10 μL) after being rinsed with cold PBS (according to the manufacturer’s protocol). Finally, the cell cycle was analyzed by Beckman CytoFLEX FCM (Bio-Rad Laboratories, Inc., Rishon Le Zion, Israel).

### 2.8. In Vivo Drug Accumulation and Tumor Growth Inhibition

Animal protocols were approved by the Institutional Animal Care and Use Committee at Ariel University. NOD/SCID (nude) mice were injected with H1299 in the right flank and K562 cells in the left flank (10^6^ cells each), so that each mouse developed dual H1299 and K562 xenografts.

When tumors reached~50 mm^3^, mice were injected via tail vein (i.v.). To investigate the peptide accumulation in the resected tumors and organs, mice were once administered with 10 mg/kg of P6-FITC [26], while for the study of the tumor growth inhibition, mice were injected once a week (a total of 3 times) with 10 mg/kg of P6-SN38, 10 mg/kg of Erbitux, or 3 mg/kg of free SN38.

### 2.9. Imaging

The H1299 (EGFR+) and K562 (EGFR−) tumors and mice organs (liver, kidney, spleen, and lung) were resected at 24 h post injection of P6-FITC, and the combined white-light and fluorescence images were taken using a fluorescence imaging system IVIS SpectrumCT (PerkinElmer). The fluorescence imaging settings were as follows: excitation filter 500 nm, emission filter 540 nm, exposure time 3 s, binning 8, and F-stop 2.

### 2.10. In Vivo Tumor Growth Inhibition

The mice’s weight and tumor volumes were measured every 2 days. The tumor sizes were measured by using the hands-on digital caliper method, and the tumor volumes were calculated according to the Formula (1) [39].

Tumor volume (mm^3^) = 0.5 × Tumor Length × Tumor Width^2^ (1)

### 2.11. Statistical Analysis

All experiments were performed in triplicate, and the results are presented as the mean ± standard deviation (SD). Data analysis was conducted using GraphPad Prism 10.0 (GraphPad Software, Inc., La Jolla, CA, USA). Statistical significance was determined using a two-way ANOVA, with a *p*-value of <0.05 considered indicative of a statistically significant difference.

## 3. Results and Discussion

### 3.1. Synthesis of P6-SN38 Peptide–Drug Conjugate

**Scheme 1:**
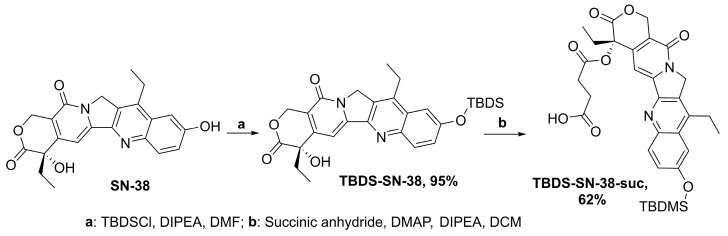
Synthesis of TBDMS-SN38-suc intermediate.

The synthetic pathway was initiated using commercially available SN38. In the initial step, the phenolic hydroxyl group of the drug was protected with a silyl ether, following the procedure outlined in the literature [38]. Subsequently, the obtained derivative, TBDMS-SN38, was reacted with succinic anhydride in DCM in the presence of DIPEA and DMAP, leading to the formation of the 20-O-succinic acid ester derivative TBDMS-SN38-suc with a reasonable yield of 62% (Scheme 1).

The synthesis of NH_2_-GABA-P6 was performed according to the previously reported procedure [26]. Accordingly, the linear peptide was first synthesized by solid-phase peptide synthesis on Rink amide resin using the standard Fmoc protocol [40]. The cyclization between the side chains of two cysteine amino acids in the linear peptide was carried out using I_2_ in DMF/H_2_O (4/1, *v*/*v*) for 2 h. Fmoc-GABA was conjugated to the Fmoc-deprotected N-terminus of the cyclic peptide residue using the PyBOP coupling reagent (Merck, Rehovot, Israel). The subsequent deprotection of the Fmoc protecting group of GABA tether led to the resin-bound NH_2_-GABA-P6.

**Scheme 2:**
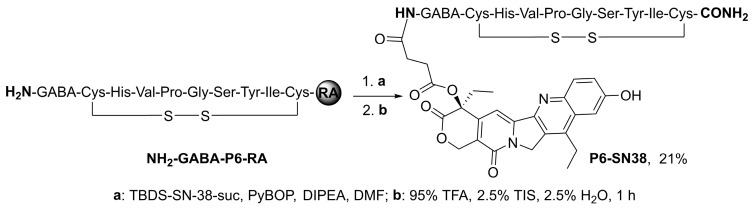
Solid phase synthesis of P6-SN38 peptide conjugate. The N and C terminus of peptide are highlighted with bold.

In the subsequent synthetic step, TBDMS-SN38-suc was conjugated with the resin-bound disulfide-bridged cyclic (oxidized) peptide NH_2_-GABA-P6 immobilized on Rink Amide (RA) resin using PyBOP as the coupling reagent in DMF with DIPEA. Notably, the side chain functional groups of the amino acids (His, Ser, and Tyr) were protected by corresponding acid-sensitive protecting groups (His with Boc and Ser and Tyr with t-Bu ether). The obtained resin-bound peptide–drug conjugate was cleaved from the solid support, with simultaneous removal of the silyl ether and amino acid side chain protecting groups, using a cocktail containing 95% TFA, 2.5% water, and 2.5% TIS. Purification by preparative HPLC resulted in P6-SN38 (purity > 95% according to LC/MS, 254 nm) with a yield of 21% (Scheme 2). The final PDC was used further for biological experiments.

### 3.2. The Effect of P6-SN38 on the Proliferation of NSCLC Cell Line

By using fluorescence microscopy and flow cytometry with the P6-FITC conjugate (P6 fluorescently labeled with fluorescein isothiocyanate), we recently demonstrated that P6 is highly specific to EGFR [26]. P6-FITC binds readily to H1299 cells expressing EGFR, but not to the HEK293 and K562 cells lacking these receptors. Also, that study showed that the conjugation of P6 with FITC did not affect its specificity. Here, taken into account the high specificity of P6 to EGFR+ cells, we examine the P6 specificity and targeted cytotoxicity of P6-SN38 in a non-small cell lung cancer (NSCLC) model.

To estimate the targeted cytotoxicity, as a negative control for the in vitro experiment, instead of non-adhesive K562 cells, we used adhesive HEK293 cells, which are more convenient in the experiments, including incubation and washing steps. Notably, both cell lines were previously utilized in ref. [26] as EGFR negative cell line controls.

**Figure 2:**
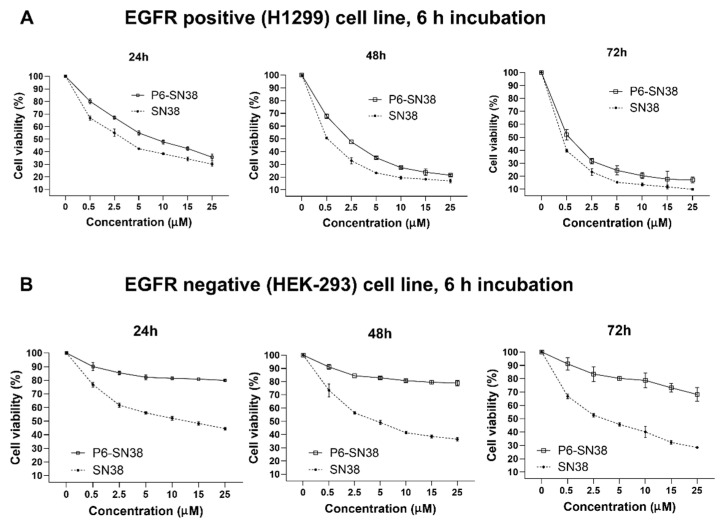
Cell viability after 6 h of pre-incubation of EGFR+ H1299 (**A**) and EGFR− HEK293 (**B**) cell lines with P6-SN38 and SN38. H1299 and HEK293 cells were cultured in a 96-well plate and pre-incubated with six different concentrations (0.5, 2.5, 5, 10, 15, and 25 μM) of P6-SN38 or SN38 for 6 h, washed out, and incubated additionally for 24 h, 48 h, and 72 h. Cell viability was assessed using an XTT assay, with the viability of untreated control cells set at 100%. The viability of treated cells was expressed as a percentage relative to the control. Data are presented as the mean ± standard deviation (SD).

Figure 2 illustrates the inhibition rates of different concentrations of the P6-SN38 conjugate in EGFR+ NSCLC cells (H1299) and EGFR− (HEK293) cells compared to the free SN38 drug. We explored two different pre-incubation times, 3 h and 6 h, and found that 3 h pre-incubation with the tested compounds was insufficient to reach meaningful results (see Figure S1), and therefore the pre-incubation time was increased to 6 h. It was found that the P6-SN38 conjugate, after pre-incubation of 6 h and subsequent washing, exhibited a noticeable dose-dependent inhibition effect on the H1299 cells after an additional 72 h of incubation, with similar sub-μM IC_50_ values as for the free SN38 (*p* < 0.05) (Table 1). On the other hand, P6-SN38 had nearly no effect on EGFR− HEK-293 cells in contrast to the free SN38, which was active in both cell lines.

### 3.3. P6-SN38 Induces Apoptosis in H1299 Cells

**Figure 3:**
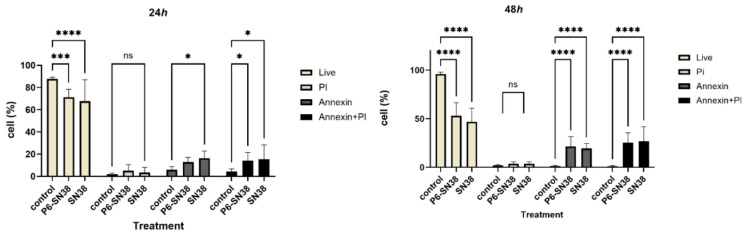
Apoptosis of H1299 cells estimated through cell survival percentage by binding with annexin V-FITC. H1299 cells were pre-incubated with P6-SN38 and SN38 as described in Section 2.6. Cells were stained with annexin and PI and analyzed by flow cytometry; * *p* < 0.05, *** *p* < 0.001, **** *p* < 0.0005. “ns” means non-significant.

SN38, a potent topoisomerase I inhibitor, disrupts DNA replication, leading to cell cycle arrest and apoptosis [41,42]. To evaluate and compare the effects of peptide-bound SN38 (P6-SN38) and free SN38 on DNA damage and cell cycle distribution (Section 3.4), H1299 cells were pre-treated with both compounds at a concentration of 2.5 µM for 6 h, followed by washing and continued incubation for 24 or 48 h (Figure 3).

Apoptosis was assessed using Annexin V-FITC/PI double staining, with flow cytometry analysis revealing similar apoptotic responses between the two compounds (Figure 3). After 24 h of exposure, the apoptotic rate induced by P6-SN38 was 12.9 ± 4.2%, while that of free SN38 was 16.3 ± 6.4%, both markedly higher than the negligible apoptosis observed in the untreated control. The late-stage apoptosis rates were also comparable between P6-SN38 (14.1 ± 7.3%) and SN38 (15.2 ± 6.8%).

After 48 h of incubation, both compounds exhibited increased apoptotic effect: 21.48 ± 9.90% and 19.27 ± 5.11% apoptosis for P6-SN38 and SN38, respectively. Consistently, the late apoptosis rates increased to 25.32 ± 10.00% for P6-SN38 and 26.80 ± 14.90% for SN38. Statistical analysis confirmed differences in apoptosis rates between treated and control groups: P6-SN38 (*p* < 0.05) and SN38 (*p* < 0.001) both significantly induced apoptosis compared to the control, with no statistically significant difference between the two treatments themselves.

The similar results presented by both compounds suggest that the binding of SN38 to the peptide P6 in P6-SN38 did not affect its apoptotic activity compared to a free SN38 in H1299 cells.

### 3.4. Increased S/G2 Cell Cycle Arrest Is Observed in NSCLC Cells upon Exposure to P6-SN38

**Figure 4:**
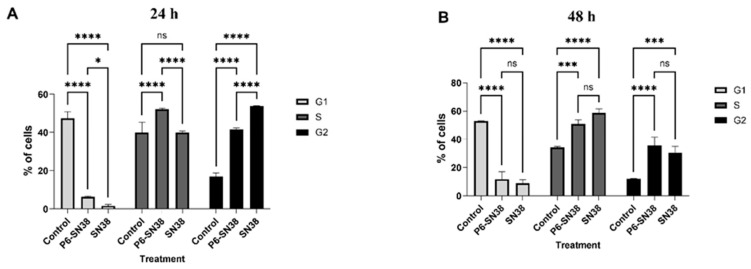

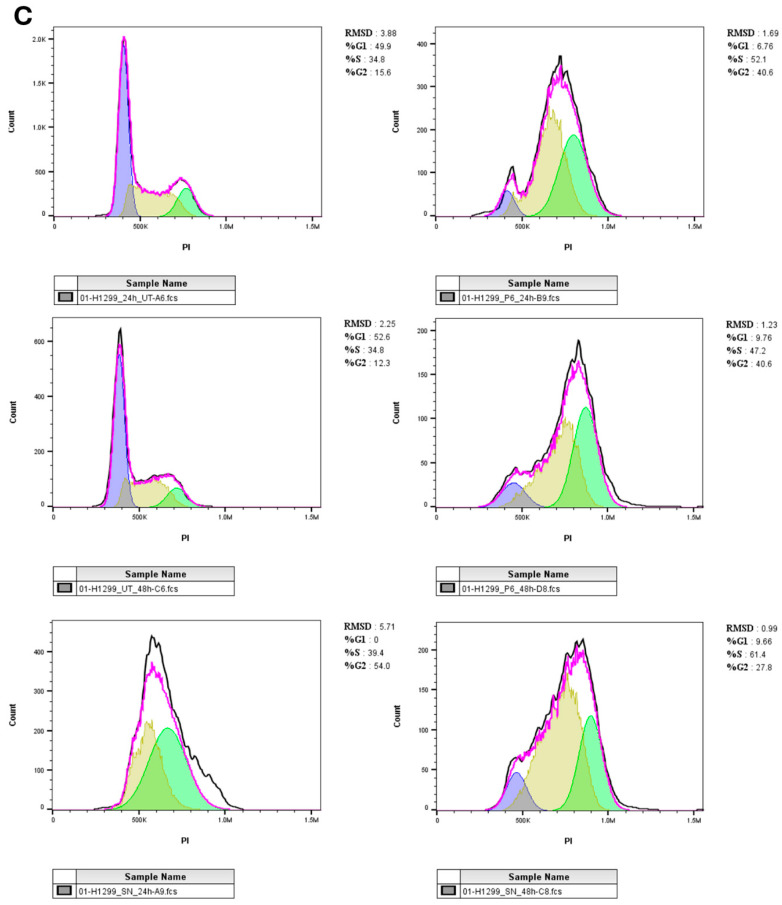
Cell cycle arrest analysis of treatment with SN38 and P6-SN38. Cell cycle distribution of H1299 cells detected by flow cytometry after 24 h (**A**) and 48 h (**B**) treatment with SN38 and P6-SN38 at 2.5 µM. A total of 1 × 10^6^ cells were pre-incubated with P6-SN38 or SN38 for 6 h, followed by additional incubation for 24 h and 48 h. The cells were then stained with propidium iodide (PI) and analyzed by flow cytometry (**C**). The data in A and B are presented as the mean ± SD (*n* = 3). “ns” means non-significant. * *p* < 0.05, *** *p* < 0.001, **** *p* < 0.0005. “ns” means non-significant.

To investigate whether the observed growth inhibition and apoptosis were mediated by cell cycle arrest, we examined the effects of P6-SN38 and free SN38 on cell cycle progression in NSCLC H1299 cells. The cells were pre-treated with the compounds for 6 h, followed by an additional 24 or 48 h incubation period (Figure 4A,B). Flow cytometry analysis revealed a notable arrest in the S phase as early as 24 h post-treatment, with a differential effect between the two compounds.

Free SN38 induced a G2/M phase arrest at 24 h, suggesting its role in inhibiting mitotic progression. In contrast, P6-SN38 caused a more pronounced arrest, affecting both the S and G2/M phases. This dual-phase arrest indicates that P6-SN38 not only interferes with DNA synthesis but also hinders progression into mitosis. After 48 h, S/G2 phase arrest was observed for both compounds, indicating sustained disruption of the cell cycle, particularly in DNA synthesis and division checkpoints (Figure 4C).

These results suggest that the cytotoxic effects of P6-SN38 may be attributed to its ability to induce more widespread cell cycle arrest, contributing to its efficacy in inhibiting cancer cell proliferation and inducing apoptosis.

### 3.5. Specific Accumulation of P6-FITC in Side-by-Side NSCLC/K562 Xenograft

Motivated by P6-SN38’s in vitro cytotoxicity and before conducting in vivo efficacy experiments, we examined its fluorescent counterpart P6-FITC for intra-tumoral accumulation in vivo.

**Figure 5:**
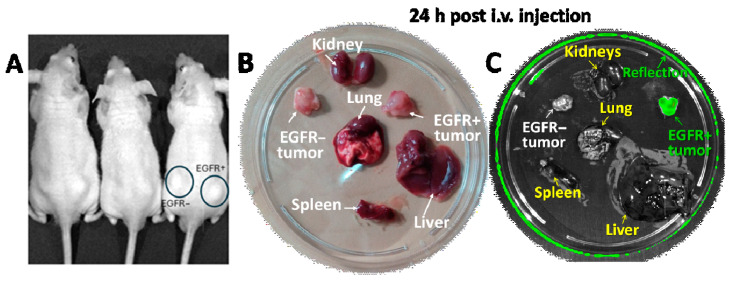
Representative side-by-side xenograft bearing K562/NSCLC tumors before injection (**A**), white light image of resected tumors and organs at 24 h post-injection (**B**), fluorescence image of resected tumors and organs at 24 h post-injection (**C**).

For that purpose, we used a dual, side-by-side EGFR+/EGFR- (H1299/K562 cell lines, respectively) xenograft nude mouse model (Figure 5A). P6 was labeled with fluorescein isothiocyanate (FITC) using the reported procedure [26] to afford the fluorescent P6-FITC conjugate. P6-FITC (10 mg/kg) was intravenously (i.v., via tail vein) injected into mice bearing side-by-side H1299 (EGFR+) and K562 (EGFR−) tumors. The tumors and organs were resected 24 h post-injection and subsequently imaged. (Figure 5B,C). A strong fluorescence signal was observed in the EGFR+ tumor (Figure 5C), whereas no signal was detected in the EGFR− counterpart and mice organs (liver, kidney, spleen, and lung). This observation provides evidence for the high specificity of the P6-FITC towards the H1299 (EGFR+) tumor. After verifying its specific intra-tumoral accumulation in H1299-derived tumors, we moved to the efficacy study with cytotoxic P6-SN38 in the same side-by-side animal model.

The fluorescence signal is observed only in the H1299 (EGFR+) tumor, evidencing high-specific accumulation of P6-FITC in the EGFR+ cells.

### 3.6. Selective Growth Inhibition of P6-SN38 in EGFR + NSCLC Xenograft

**Figure 6:**
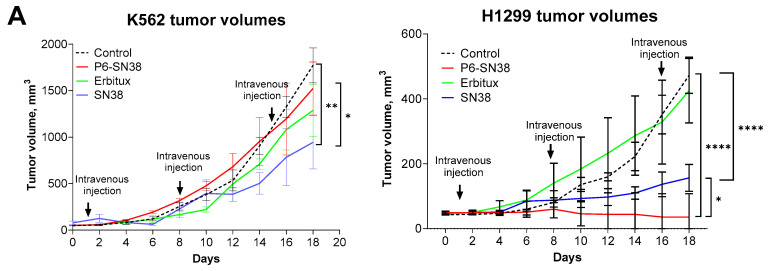

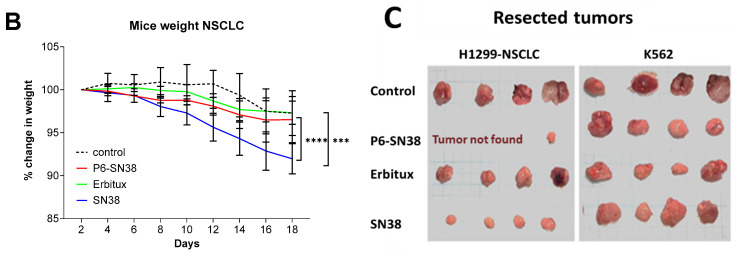
In vivo effect of P6-SN38 conjugate in NSCLC model (10 mg/kg, once a week). Effect of P6-SN38, free SN38, and Erbitux on tumor volume compared to non-treated control (**A**) and mice weight (**B**). The resected tumors are shown in (**C**). *N* = 4 in each group. * *p* < 0.05, ** *p* < 0.005, *** *p* < 0.001, **** *p* < 0.0001.

After estimating targeted accumulation of fluorescent P6-FITC in NSCLC xenograft (Section 3.5), we evaluated the efficacy of its cytotoxic counterpart P6-SN38 in the same side-by-side mouse model bearing K562/NSCLC tumors (Figure 6). During the study, the efficacy of P6-SN38 was compared to that of free SN38, the anti-EGFR antibody Erbitux, and untreated control.

The H1299 tumor did not exhibit statistically significant growth in tumor size when compared to the control group in pairwise comparisons conducted before drug administration and three and six days after administration (*p* > 0.05). During the study, the H1299 tumors (right dorsal) in the PDC-injected mice do not significantly grow over the course of the experiment; instead, they gradually shrink after seven days (Figure 6A). By the 18th day, H1299 tumors in the untreated control group reached a volume of 476 ± 55 mm^3^, and the K562 tumors in the same control group reached 1772 ± 265 mm^3^, demonstrating a 10-fold and 30-fold volume increase, correspondingly, compared to their initial volume (~50 mm^3^). The K562 tumors of the PDC-injected group reached a volume of 1522 ± 561 mm^3^, similar to that of the control. However, the H1299 tumor in the PDC-treated group was eradicated completely in 3 out of 4 mice (Figure 6C). Thus, the P6-SN38 conjugate caused specific EGFR+ NSCLC eradication but did not affect the growth of K562 (EGFR−) tumors. Furthermore, P6-SN38 was more successful in animals than free SN38 in shrinking NSCLC. Animals treated with P6-SN38 also showed minimal weight loss compared to the control and free SN38 groups (Figure 6B), suggesting the potential safety and reduced toxicity of the P6-SN38. The assumption that P6-SN38 induces lower toxicity in comparison to the free payload is supported by this observation. Free SN38, however, showed non-specific tumor reduction in treated mice with significant weight loss, pointing to toxicity induction. Erbitux was ineffective at inhibiting tumor growth, but it did cause negligible weight loss. Due to the K562 tumor’s excessive size, the experiment was stopped on day eighteen in accordance with ethical regulations.

## 4. Conclusions

We synthesized a novel peptide–drug conjugate, P6-SN38, that targets EGFR overexpressed NSCLC. It consists of EGFR specific peptide ligand P6 connected through the mono-succinate linker to the potent Topo I inhibitor SN38. P6-SN38 showed selective cytotoxicity to EGFR-positive NSCLC cells in vitro but no effect on EGFR-negative HEK-293 cells. In terms of cell cycle progression, P6-SN38 induced cell cycle arrest in both the S and G2/M phases, which is consistent with the mechanism of action of the Topo I inhibitor SN38. This dual-phase arrest indicates that P6-SN38 may disrupt both DNA synthesis and mitotic progression, which could contribute to its enhanced cytotoxicity compared to free SN38. Along with cell damage and cell cycle arrest similar to the free SN38, it was also demonstrated that P6-SN38 preserved cytotoxicity in NSCLC cells in vitro. The in vivo imaging of fluorescently labeled P6-FITC in a side-by-side xenograft model of H1299/K562 (EGFR+/EGFR− cell lines, respectively) resulted in specific intra-tumoral accumulation of P6-FITC in EGFR+ NSCLC tumors. Additionally, P6-SN38’s efficacy evaluation in the same side-by-side xenograft model showed specific inhibition of NSCLC growth, indicating targetability to tumors overexpressed for EGFR. In contrast to mice treated with free SN38, mice treated with P6-SN38 exhibited a very moderate weight decrease, suggesting tolerable side effects. According to the results obtained in this study, the developed P6-SN38 conjugate can effectively and selectively inhibit the growth of EGFR+ NSCLC in vivo. We therefore anticipate that this PDC can be successfully utilized not only for the treatment of non-small cell lung cancer but also for other cancers with intracellular EGFR mutations.

## Figures and Tables

**Table 1:** IC_50_ values of P6-SN38 and SN38 on H1299 and HEK293 cells after 6 h of pre-incubation.

	IC_50_ (μM)			
	H1299		HEK293	
Incubation time	48 h	72 h	48 h	72 h
SN38	0.8	0.5	4.4	2.7
P6-SN38	2.5	0.9	>50	>50

## Data Availability

The original contributions presented in this study are included in the article/Supplementary Materials. Further inquiries can be directed to the corresponding authors.

## Supplementary Materials

The following supporting information can be downloaded at: https://www.mdpi.com/article/10.3390/pharmaceutics16121613/s1, Figure S1: In vitro experiment of P6-SN38 after 3 h preincubation.
