# Supplementary material for: Novel Cyclic Peptide–Drug Conjugate P6-SN38 Toward Targeted Treatment of EGFR Overexpressed Non-Small Cell Lung Cancer
Source: Pharmaceutics. 2024 Dec 19;16(12):1613. doi: 10.3390/pharmaceutics16121613 (PMC11676734; doi:10.3390/pharmaceutics16121613)

Supporting Information

Novel Cyclic Peptide Conjugate P6-SN38 Toward Targeted Treatment of EGFR Overexpressed NSCLC

1. XTT assay

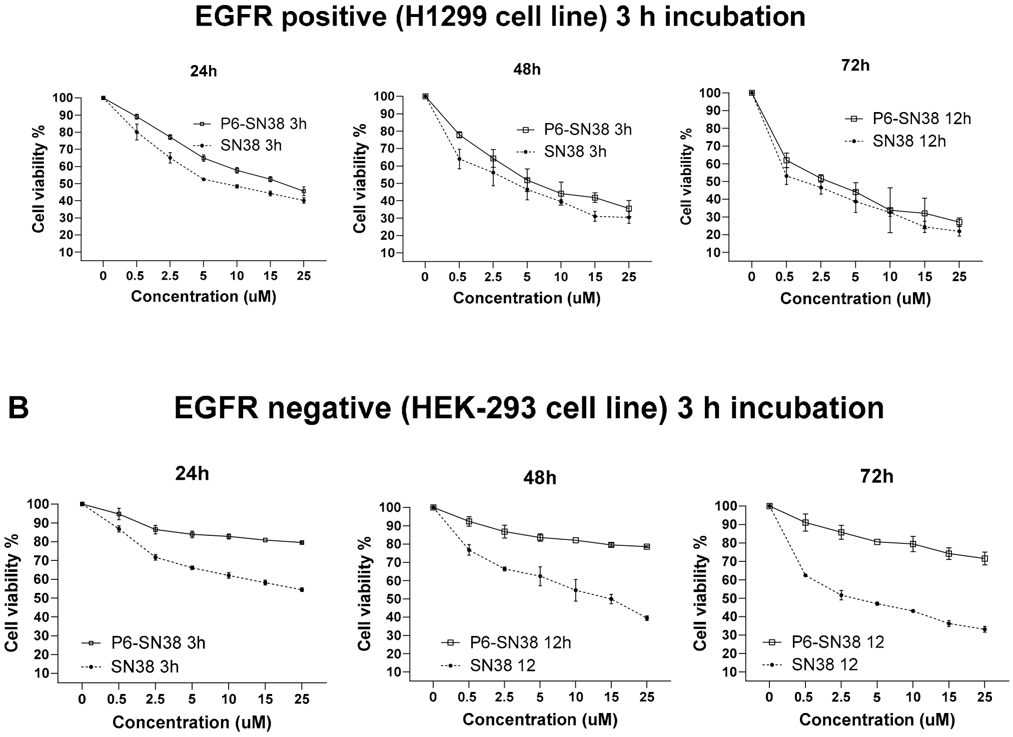

Figure S1: In vitro experiment of P6-SN38 after 3 h preincubation

2. ^{1}H & ^{13}C NMR, LCMS and HRMS Spectra

<u>TBDS-SN-38-Suc</u>

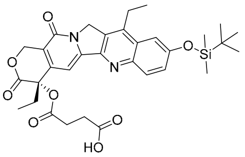

TBDS-SN-38-Suc,
Chemical Formula: $C_{32}H_{38}N_2O_8Si$
Exact Mass: 606.24

Supporting Information

^{1}H NMR

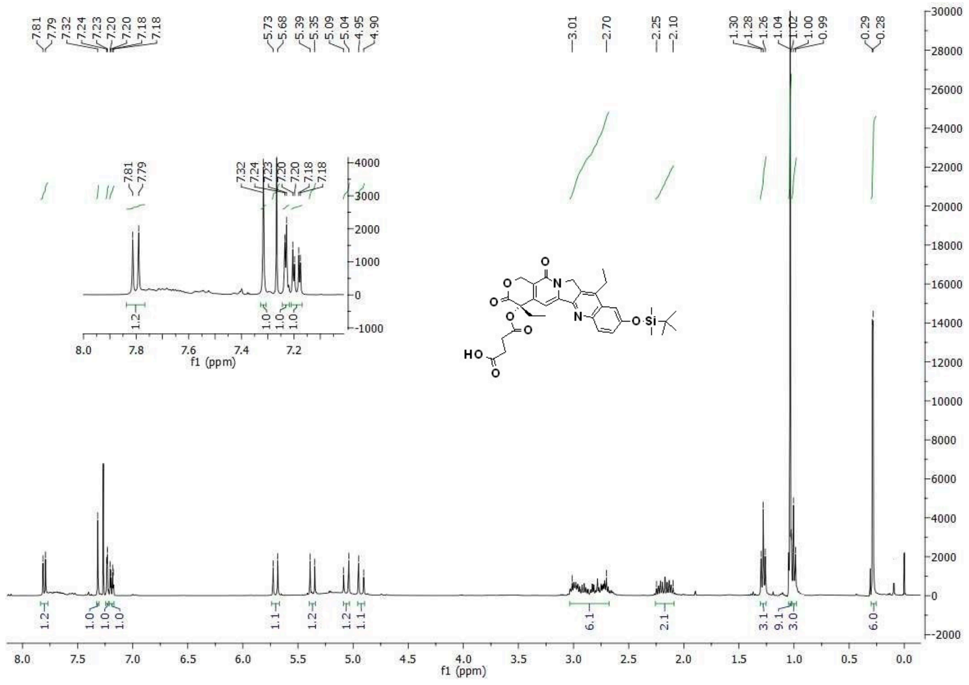

^{13}C NMR:

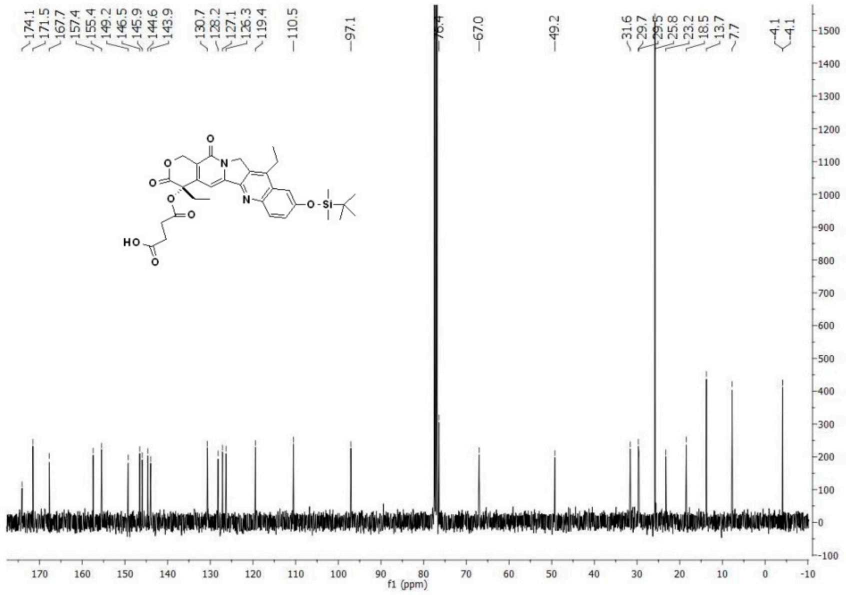

Supporting Information

HRMS:

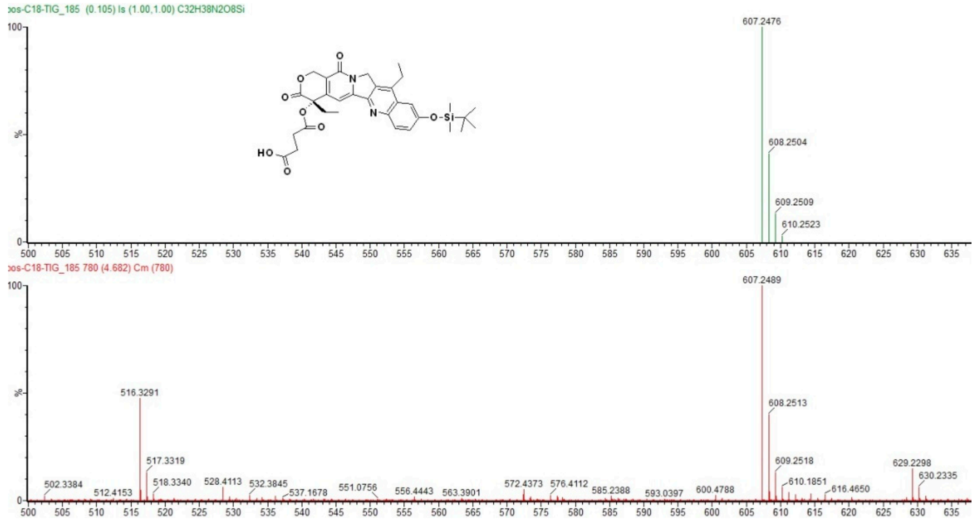

<u>P6-SN38</u> <u>(P6-GABA-Suc-SN-38)</u>:

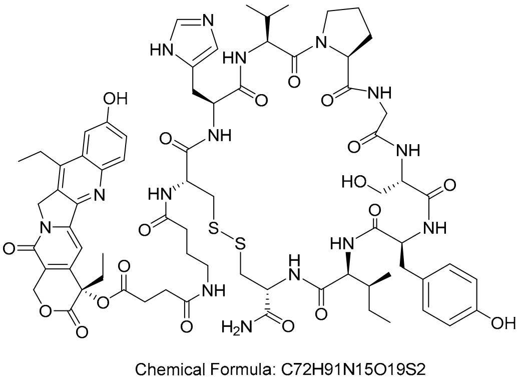

Chemical Formula: C72H91N15O19S2
Exact Mass: 1533.61
$(M+2H)^{2+}$= 767.8

Supporting Information

LCMS:

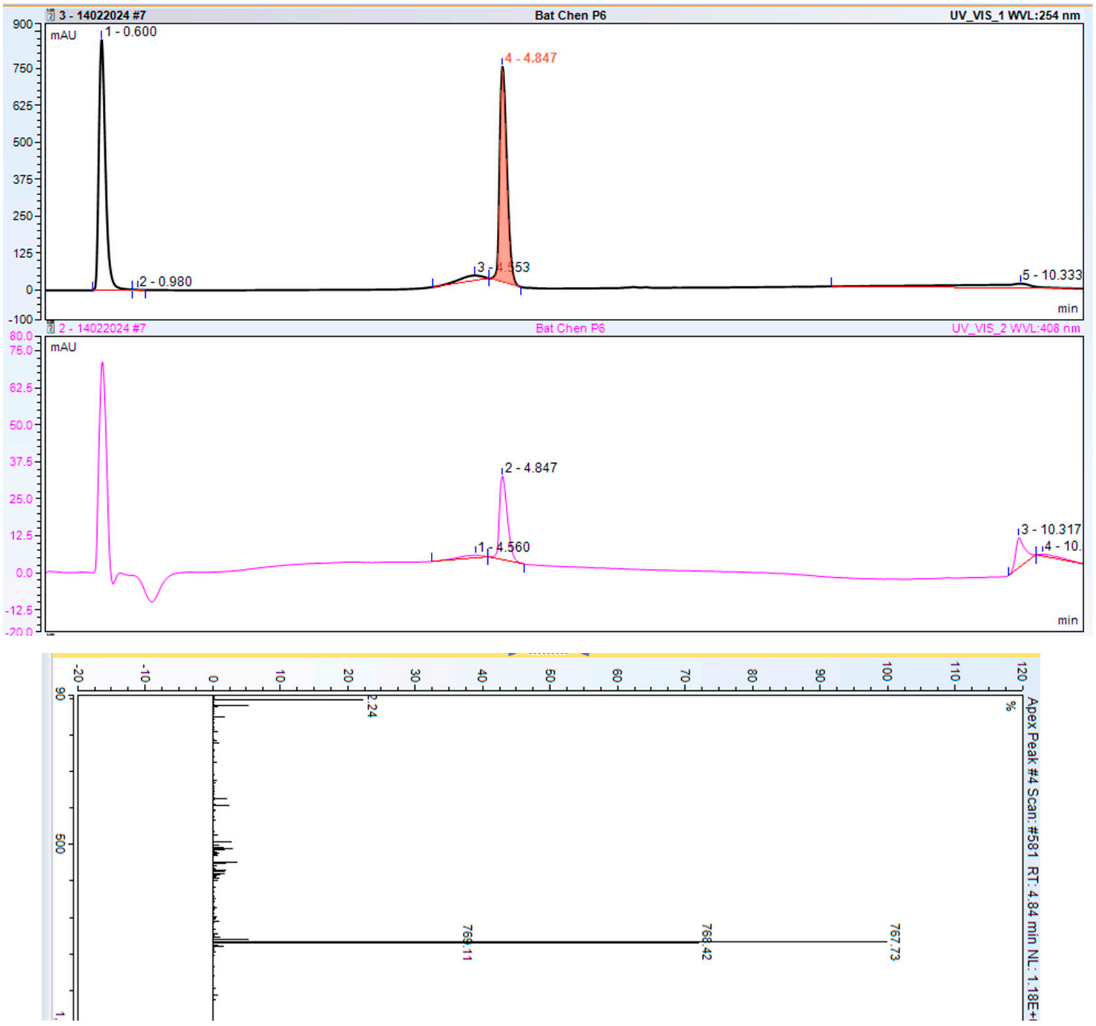

Supporting Information

HRMS:

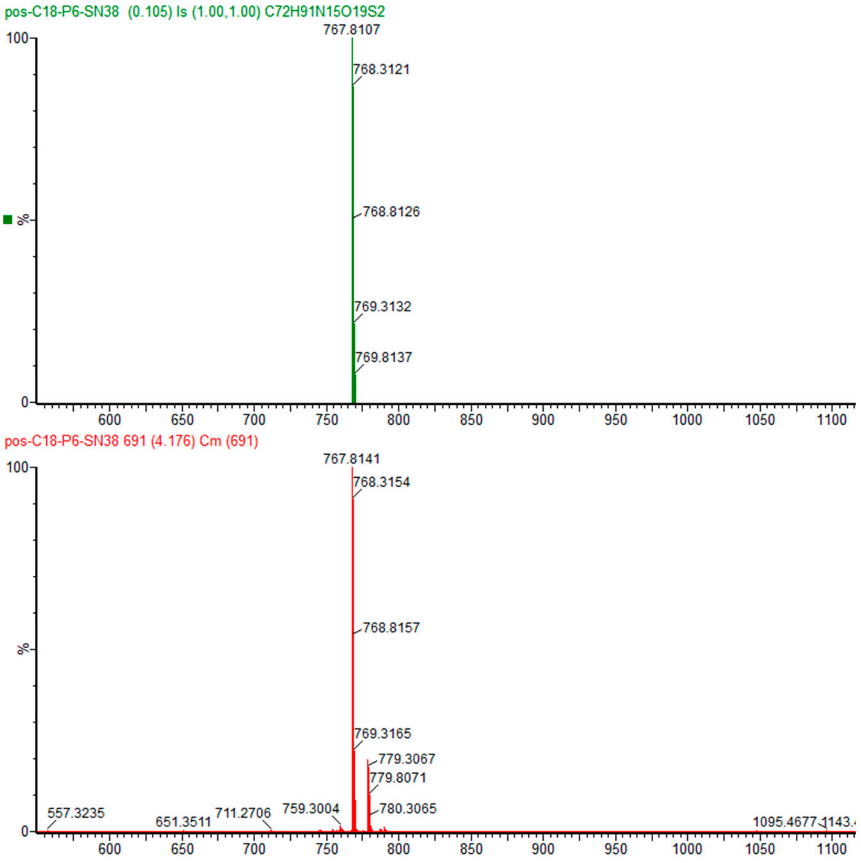

Supplement: Supplementary file 1 [file pharmaceutics-16-01613-s001.zip › pharmaceutics-3339315-supplementary.pdf]
